# Influence of a Municipal Solid Waste Landfill on the Surrounding Environment: Landfill Vegetation as a Potential Risk of Allergenic Pollen

**DOI:** 10.3390/ijerph16245064

**Published:** 2019-12-12

**Authors:** Magdalena Daria Vaverková, Dana Adamcová, Jan Winkler, Eugeniusz Koda, Jana Červenková, Anna Podlasek

**Affiliations:** 1 Department of Applied and Landscape Ecology, Faculty of AgriSciences, Mendel University in Brno, Zemědělská 1, 613 00 Brno, Czech Republic; dana.adamcova@mendelu.cz; 2 Institute of Civil Engineering, Warsaw University of Life Sciences–SGGW, Nowoursynowska 159, 02 776 Warsaw, Poland; eugeniusz_koda@sggw.pl (E.K.); anna_podlasek@sggw.pl (A.P.); 3 Department of Plant Biology, Faculty of AgriSciences, Mendel University in Brno, Zemědělská 1, 613 00 Brno, Czech Republic; jan.winkler@mendelu.cz (J.W.); xcerven3@node.mendelu.cz (J.Č.)

**Keywords:** municipal solid waste, ecological regeneration, revegetation, allergenic pollen

## Abstract

When the landfill use comes to end, important subsequent steps include aftercare, safety assurance, and ecological regeneration. Landfill revegetation is cost-effective and eco-friendly approach in the management of landfill areas, which serves the purpose of stabilization and provides a pleasant landscape. There are various vegetation types that can be planted, yet grass species are often used for low-cost reasons. Plants can be important sources of air pollution, particularly by grass pollen. The main goal of our study was to identify plant species that produce allergenic pollen. Long-term vegetation monitoring took place on three sites in the growing seasons of years 2008–2018. Studied objects were landfills located in the Czech Republic. The vegetation was assessed using a floristic survey of identified plant species. Plant species that produced allergens were recorded. During the monitoring, 298 plant species were determined. Plant species with allergenic pollen have a considerable share in the landfill vegetation. Thus, landfills are potential sources of various kinds of allergenic pollen. Moreover, our results indicated that there are three periods of pollen production: early spring, late spring, and early summer; late summer; and autumn. The second period is typical for the production of highly allergenic pollen by grasses. Most detected plant species with allergenic pollen are common for all monitored sites, which demonstrates that the vegetation of landfills is a significant source of allergenic pollen.

## 1. Introduction

The disposal of municipal solid waste (MSW) in landfills entails a number of environmental risks and raises concerns about harmful impacts on human health, e.g., pollution of air, soil, and groundwater, risk of fires and explosions, bad odors, or damage to vegetation [1,2,3,4]. During the landfilling, a wide range of pollutants is released into the environment, especially in to the atmosphere: landfill gases and particular matter (PM). Landfill gas emissions contain a large number of compounds of which the most important are methane (CH_4_), carbon dioxide (CO_2_), and various mixtures of pollutants including volatile organic compounds (VOC). PM emissions may have toxic properties as they reflect the chemical composition of the disposed waste [1,2,3,4]. When the landfill use comes to end, important subsequent steps include aftercare, safety assurance, and ecological regeneration. Regeneration is a process that will return a site to a condition suitable for its proposed after use whilst incorporating measures to protect human health and the environment [5]. Recovery of impaired ecosystems in landfill sites needs a sensitive and correct approach. In urbanized areas, such restored localities can serve as sites for the enhancement of biological diversity [6,7,8]. During the reclamation of landfill body surface, arable land is as a rule grassed to prevent water or wind erosion of its slopes. There are various vegetation types that can be planted (grasses, herbs, shrubs, or trees), depending on the landfill terrain reclamation—phytocapping of landfills [9,10,11]. Phytocaps can provide a cost effective and sustainable alternative when compared with traditional top barriers. Obvious advantages are their lower cost, utilization of available resources (use of local soils and native plants), high ecological site improvement, and potential of greenhouse emission reduction [12]. The selection of plant species relies on the adaptability of the chosen species to local climate and soil substrate [9]. However, grass species (e.g., *Lolium perenne*, *Festuca rubra*, *Festuca ovina*, *Festuca pratensis*, *Arrthenatherum elatius*, *Poa pratensis*, *Cynosurus eristatus*, *Bromus inermis*, and *Bromus erectus*) are usually used for low-cost reasons. 

Plants used to reclaim landfill bodies can be important sources of air pollution namely by atmospheric emissions of grass pollen. The pollen of grass species pollinated by wind can represent a significant health risk because of its high allergenic potential [13]. These grass species are considered the main cause to pollen allergies in many parts of the world [14,15,16]. Airborne pollen concentrations have traditionally been used to establish potential exposure to airborne allergens. Nevertheless, research has shown that allergy symptoms may arise even at low pollen concentrations [17]. Moreover, there are not only allergens linked to pollen grains and non-pollen-bound allergenic substances but also free allergens that can be also found in the atmosphere [14,18]. Recently, the role of grass pollen in respiratory allergy was confirmed in the European Community Respiratory Health Study [17,19]. The overall prevalence of seasonal allergic rhinitis in Europe and the United States is approximately 15%. Sensitization to pollen allergens is increasing in most developed countries and synergism with other common atmospheric pollutants has also been identified, though the mechanisms of interactions have not been fully understood [20]. This requires accurate forecasting of atmospheric levels of these pollutants. Modeling of pollen emissions and dispersion remains a challenging task [21,22], and currently there are no methods established for modeling ambient concentrations, population exposures, and doses of pollen and co-occurring aeroallergens. While it is generally accepted that most pollen registered by observational networks comes from local sources [23], there is also a growing evidence of long-range transport of pollen [24,25,26].

In the pollen season, grasses exude pollen in high concentrations, which leads to allergic symptoms starting from seasonal *rhinoconjunctivitis* up to *asthma bronchiale* in susceptible individuals [27,28]. Moreover, the pollen of some plants commonly used in landfill reclamation such as birch (*Betula*) and sagebrush (*Artemisia*) rank with the most significant aeroallergens [25].

Our research team is involved in the long-term monitoring of environmental problems of pollutants produced and released from landfills (e.g., landfill gas, leachate, biomonitoring). This research was devoted to vegetation monitoring and identification of the share of plant species that produce allergenic pollen on three MSW landfill sites located in the Czech Republic (CR). In each detected plant species, we determined the time of its blooming, mode of pollen transfer, and importance of pollen as human allergen. Based on these data, plant species and seasons (months) potentially problematic with respect to the production of allergenic pollen were determined.

Because the most common method of landfill reclamation in CR is grassing (e.g., *Lolium perenne*, *Festuca rubra*, *Festuca ovina*, *Festuca pratensis*, *Arrthenatherum elatius*, *Poa pratensis*, *Cynosurus eristatus*, *Bromus inermis*, and *Bromus erectus*) the authors hypothesized that the vegetation of MSW landfill can be a significant source of allergenic pollen.

The hitherto published scientific studies mainly deal with the direct adverse impact of MSW landfills on the environment (leakage of hazardous substances, formation of leachates, landfill gas, etc.) or with their technical and biological reclamation. Research of landfill vegetation and possible production of pollen have been neglected so far despite the fact that pollen production can have a negative impact on human health. Therefore, the main objectives of our research were as follows: (1) identification of plant species growing on MSW landfills, which may produce allergenic pollen, (2) determination of main seasons of allergenic pollen production on MSW landfills, and (3) proposal of procedures for the control of landfill vegetation in order to reduce or minimize production of allergenic pollen.

## 2. Materials and Methods

### 2.1. Study Area

**Figure 1:**
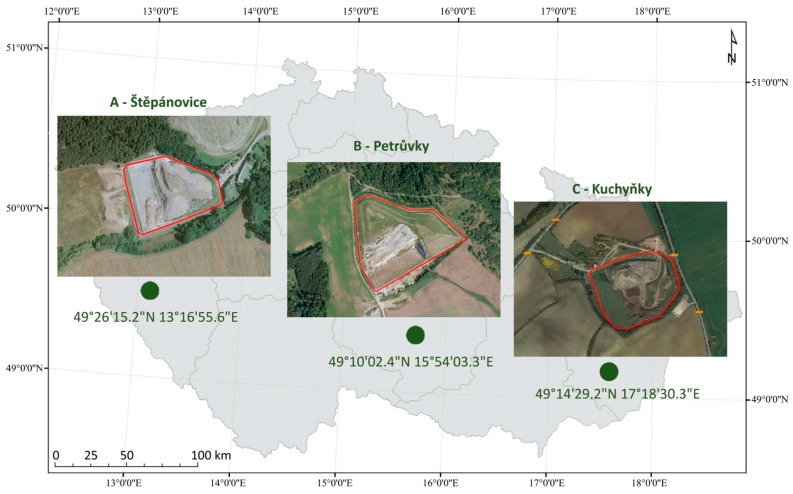
Locations of landfills in the Czech Republic.

The research was implemented on the following three sites of MSW landfills situated in the CR (Figure 1): Štěpánovice—49°26′15.7″ N 13°16′56.5″ E (A), Petrůvky—49°10′00.7″ N 15°54′03.3″ E (B), and Kuchyňky—49°14′29.7″ N 17°18′22.8″ E (C). All three landfills are modern and sanitary landfills, established for waste disposal and storage at or under the terrain level, Code D1 pursuant to valid legislation (Ministry of the Environment of the CR, 2019). Moreover, these landfills are located around the borders of municipalities, far away from the central business districts (CBD) and residential zones. A brief characteristic of selected localities with MSW landfill sites is presented in Table 1.

The large Kuchyňky landfill site is situated in an undulating and open agricultural landscape. The chosen form of biological reclamation was grassing. The site is situated in Climatic Region 3—warm, mildly humid (T3) with the following characteristics: mean annual temperature 8–9 °C, mean annual precipitation amount 270–285 mm, moisture security in the growing season 4–7. The main soil unit consists of Haplic Chernozem, Haplic Luvisol, Albic Luvisol, Haplic Albeluvisol, Haplic Cambisol, and Albic Cambisol. Hydropedological characteristic of the region: soils with the medium infiltration rate even at full saturation are mainly medium deep to deep, medium to well drained, loamy-sand to clay-loamy soils. Medium inclination, gradient 7–12°. Soil skeleton content was up to 10%.

The Petrůvky landfill is a large size, with a meadow on the southwest. An unpaved road forms the boundary between the landfill site and the meadow. The northeastern border is lined with forest. The chosen form of biological reclamation was grassing. The Petrůvky landfill is situated in Climatic Region 5—mildly warm and humid (MT2). The region’s characteristics are as follows: mean annual temperature 7–8 °C, mean annual precipitation amount 550–650 mm, and moisture security in the growing season 4–10. The main soil unit consists of Lithic Cambisol, Haplic Cambisol, and Haplic Leptosol. Hydropedological characteristic of the region: soils with the medium infiltration rate even at full saturation are mainly medium deep to deep, medium to well drained, loamy-sand to clay-loamy soils, mild inclination, gradient 3–7°, and a soil skeleton content of 25–50%.

The Štěpánovice landfill is smaller and situated in a valley; its northern side is surrounded with forest. The chosen biological reclamation was afforestation. Similarly, as the Petrůvky landfill, the Štěpánovice landfill is situated in Climatic Region 5 with the following characteristics: mean annual temperature 7–8 °C, mean annual precipitation amount 550–650 mm, and growing season moisture security 4–10. The main soil unit consists of Haplic Cambisol eubasic and Haplic Cambisol mesobasic. The hydropedologic characteristics of the region are as follows: soils with the medium infiltration rate even at full saturation are mainly medium-deep to deep, medium to well drained, loamy-sand to clay-loamy soils, medium inclination with the gradient of 7–12°, and a soil skeleton content of 25–50%.

The landfills of Kuchyňky, Petrůvky, and Štěpánovice are in the S-OO group (other waste). Waste deposited in these landfills includes communal wastes from adjacent towns and villages. Landfill bodies are regularly extended by individuals mutually linked in subsequent stages, the procedure respecting the originally declared general master plan, which passed through the process of environmental impact assessment (EIA). The landfills were already subjected to the first stages of technical and biological reclamation. Although the reclamation works significantly mitigated impacts of these facilities on the environment, the selected type of biological reclamation may still affect the adjacent ecosystems.

### 2.2. Vegetation Study and Plant Analysis

Long-term vegetation monitoring took place in three localities (i.e., Kuchyňky, Petrůvky, and Štěpánovice) in the growing seasons (February (II)–October (X)) in 2008–2018. January, November, and December were excluded from the monitoring, because the winter period is the period of dormancy and rest of the plants.

The research sites were selected according to the method of biological reclamation—grassing at the Kuchyňky and Petrůvky landfills and grassing combined with afforestation at the Štěpánovice landfill. All monitored facilities are subject to regular collection and analyses of data from the measurement of the production of leachates, landfill gas, and the amount and type of disposed and stored wastes. Potential impact of the landfills on the environment is a subject to long-term monitoring, too [29,30,31]. From our previous research of landfill sites, it is evident that classic landfill monitoring should be supplemented with biomonitoring. The method of biomonitoring that makes use of vegetation cover is simple, fast and cost-effective. However, it is limited by the lifetime of monitored plant species. This method of assessing the vegetation has not been used much [32,33]. Plants provide a singular opportunity to explore biological effects of contamination and give reliable information about the quality and characteristics of the environment [33]. Therefore, in the present study, we analyzed vegetation by using a floristic survey of the identified plant species. Research plots were established within the surface areas of the MSW landfills. Sampling was carried out during the vegetation season (2008–2018) when most species were expected to be growing. Plant species producing allergens identified in these research plots were recorded. Names of the respective plant species were used according to Danihelka et al. [34]. Each detected plant species was classified according to: (1) blooming time, (2) pollen transfer mode, and (3) role of landfills in the production of pollen as allergen. The blooming time and the mode of pollen transfer were borrowed from Pladias [35]. The pollen producing plant species were borrowed from the database of the Czech Pollen Information Service (PIS) monitoring the occurrence of pollen and other biological pollutants in the air. The PIS data served physicians and patients for information on air quality and helped improve patients’ treatment [36].

The pollen producing plant species identified on the chosen plots were divided into four categories: 3—plant species producing allergenic pollen; 2—wind-fertilized plant species whose pollen is airborne but does not belong to strong allergens; 1—entomophilous plant species whose pollen is borne by insects and gets into the air only at limited amounts; 0—plant species with no pollen production (cryptogams). Data from the 10-year monitoring of the occurrence of individual plant species on the three landfill sites, their significance in terms of allergies, and blooming time of the detected species were processed by using the multivariate analysis of ecological data. The obtained data were analyzed by using detrended correspondence analysis (DCA) and segment analysis. Subsequently, canonical correspondence analysis (CCA) was used to express the correlation between the locality, the months of blooming, and the detected species. Furthermore, the data were processed by the Monte Carlo test, using 999 permutations and processed by using the Canoco 5.0 software [37]. Statistical hypotheses were as follows: (i) there is no correlation between the months of blooming and the landfill vegetation, (ii) the site does not affect the composition of landfill vegetation, and (iii) the year of observation has no effect on the composition of landfill vegetation.

## 3. Results and Discussion

During the monitoring, we found and determined altogether 298 plant species in all sites. Table 2 presents the number of plant species found in the respective localities, the number of plant species blooming in the assessed months (February–October) and the number of plant species evaluated according to their production of allergenic pollen.

**Figure 2:**
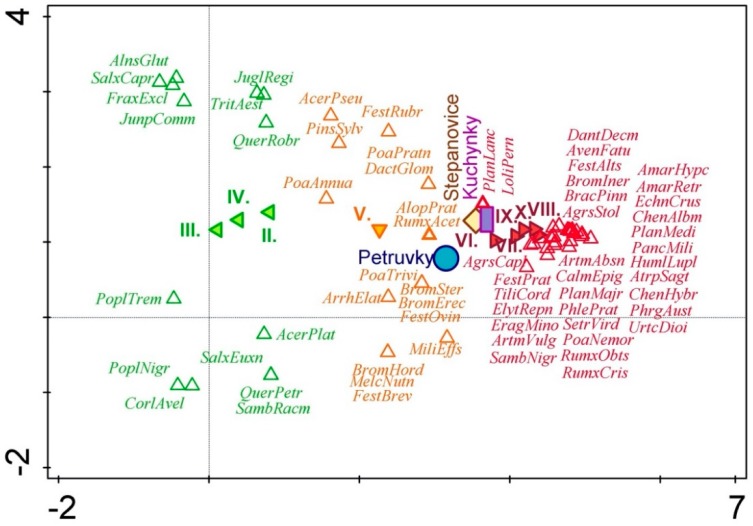
Relations among the monitored sites, blooming times and identified plant species producing allergenic pollen. Legend to abbreviated names of some plant species is presented in Appendix (Appendix A). Green: abbreviated names of detected plant species blooming mainly in the period from February to April (II–IV). Orange: abbreviated names of detected plant species blooming mainly in May (V). Red: abbreviated names of detected plant species blooming mainly in the period from June to October (VI–XX).

Result of the analyses is a spatial arrangement of processed data on the plant species, monitored sites, and months (February–September: II–X), which are illustrated as points in the ordination diagram. The closer the individual points are to one another, the tighter their relation is. If the point of a certain plant species occurs near the point of a certain month (II–X), the month can be considered as the period of blooming of that plant species (Figure 2).

The number of plant species that exhibit the occurrence of pollen (pursuant to PIS) and considered as plants producing pollen causing allergic reaction in humans totaled 65. Hence, we can state that the vegetation of MSW landfills can be a source of allergenic pollen. The highest number of such plant species was found in the Kuchyňky landfill site and the least number of them was detected in the Štěpánovice landfill site. The share of species producing allergenic pollen in the total amount of plant species was approximately 25%. The analysis showed that the representation of species producing strongly allergenic pollen is considerable in the vegetation growing on the MSW landfills. Moreover, according to Plaza et al. [18] in the case of plants, environmental changes produced by the waste discarded at these sites (organic, inorganic, metals, ash, coal, and biological waste) favor the growth of invasive species. This was also confirmed by Vaverková et al. [33].

At this place, it should be pointed out that the chosen type of biological reclamation can significantly influence the incidence of species producing allergenic pollen. The Kuchyňky landfill site is of large size, situated in an undulating and open agricultural landscape. Since the chosen form of biological reclamation was grassing, the number of plant species producing allergenic pollen was high on this landfill. The Štěpánovice landfill is smaller, situated in a valley, its northern side is surrounded with the forest and the chosen biological reclamation was afforestation. It was demonstrated that this landfill is covered with the lowest number of plant species producing allergenic pollen. After the full growth of the trees, the grass cover composition is likely to be affected.

Based on the results of segment analysis (DCA), which expresses the correlation among the site, the blooming term and the detected plant species, pollen production is obviously uneven during the growing season and the representation of individual pollens is changing. Figure 1 shows that the species can be classified into three groups according to their blooming time when they produce pollen. Ordination diagram plotted by using the DCA analysis expresses spatial relations of the occurrence of plant species and monitored factors by means of coordinates. If coordinates of the point of the given species are similar to coordinates of the selected factor (being close to each other in the figure), the relation between them is stronger. In such a case, the given species produced pollen in the respective month or was recorded in the given site more frequently.

The first group of species bloom mainly in the spring (February–April, green color in Figure 1). This group includes primarily the following trees and shrubs: *Acer platanoides*, *Alnus glutinosa*, *Corylus avellana*, *Fraxinus excelsior*, *Juglans regia*, *Juniperus communis*, *Populus nigra*, *Populus tremula*, *Quercus petraea*, *Quercus robur*, *Salix caprea*, *Salix euxina*, *Sambucus racemosa,* and *Triticum aestivum*. The species mainly represent remainders of the original vegetation, usually adult individuals blooming and producing pollen, which thus represent a certain risk in terms of increased amounts of allergenic pollen in the air. Another case are young seedlings introduced to the site with waste, which are unimportant with respect to the production of allergens and often die in the landfill environs before they grow up and start producing pollen.

**Figure 3:**
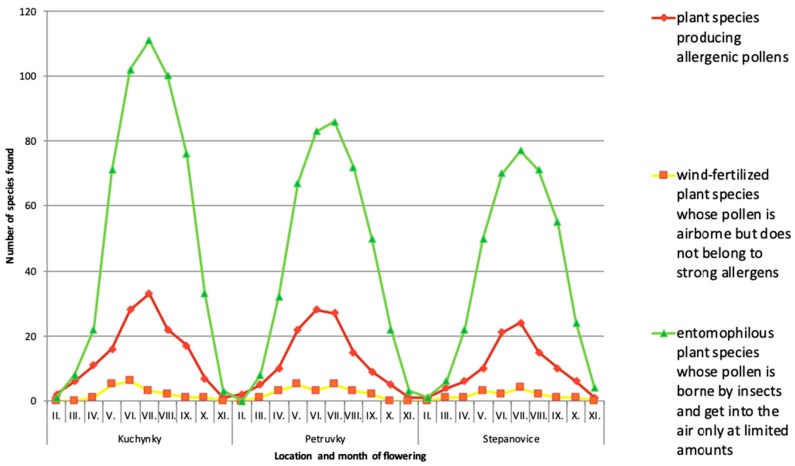
Number of blooming plant species found in the selected landfills.

The second group is represented mainly by grasses, which bloom in late spring (May, orange color in Figure 1) and includes the following grasses, herbs, and some tree species: *Acer pseudoplatanus*, *Alopecurus pratensis*, *Arrhenatherum elatius*, *Bromus erectus*, *Bromus hordeaceus*, *Bromus sterilis*, *Dactylis glomerata*, *Festuca brevipila*, *Festuca ovina*, *Festuca rubra*, *Melica nutans*, *Milium effusum*, *Pinus sylvestris*, *Poa annua*, *Poa pratensis*, *Poa trivialis,* and *Rumex acetosella*. The species primarily occur on the reclaimed and grassed surfaces of landfills and/or at places with the remainders of the original vegetation. Pollen production in a majority of them is limited by vegetation cutting. By cutting the grassy vegetation prior to blooming, the production of allergenic pollen in the late spring and at the beginning of summer can be considerably reduced. Pursuant to the normative regulation of “ČSN 83 8035 Landfilling of waste—Landfill closure and reclamation, setting guidelines for the establishment and treatment of grass stands on the landfill body” [38], the grass stands are to be cut two times a year at the least, the first cut to be implemented in May–June and the second to be done in August–September. Figure 3 shows the number of blooming plant species found in the selected landfills. As can be seen on the graph (Figure 3), the largest number of plants producing allergic pollen falls into the period from May till September (V–IX). This can be observed in all studied landfills.

**Figure 4:**
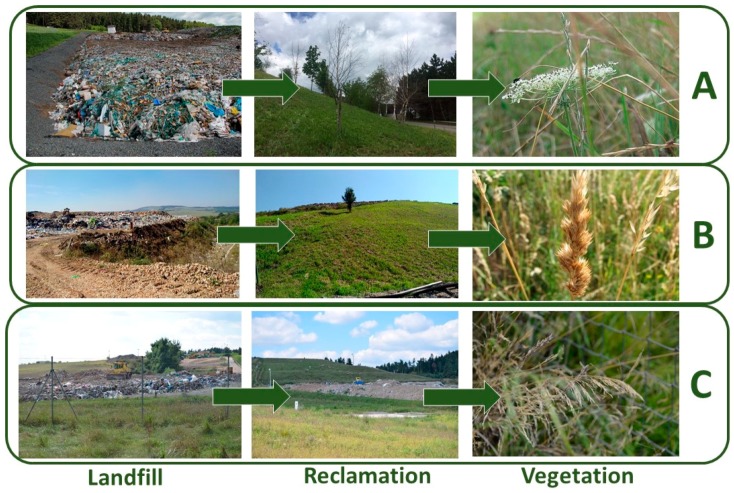
Grass stands of reclaimed landfill parts: Štěpánovice (**A**), Petrůvky (**B**) and Kuchyňky (**C**) landfill.

The grass should be taken care of in order to form a continuous grass stand (Figure 4). To minimize the occurrence of allergenic pollen, the cutting of reclaimed landfill is advised during the month of May. This finding should be implemented in legislation, which would force landfill operators to maintain the reclaimed surfaces in time and thus to contribute to the minimization of the production of strong allergens. This result highlights that although the forest reclamation is more expensive and technically demanding [39], it could significantly contribute to the enhancement of air quality, being more appropriate in the landscape, creating a better microclimate, and helping to increase biological diversity in the given site. Moreover, forest reclamation reduces the growth of pollen producing plants and grasses. These aspects should be taken into account in creating the normative and legislative environment.

The third group includes the following mainly annual and perennial herbs blooming in the period from summer to autumn (June–October, red color on Figure 1): *Agrostis capillaris*, *Agrostis stolonifera*, *Amaranthus hypochondriacus*, *Amaranthus retroflexus*, *Artemisia absinthium*, *Artemisia vulgaris*, *Atriplex sagittata*, *Avena fatua*, *Brachypodium pinnatum*, *Bromus inermis*, *Calamagrostis epigejos*, *Danthonia decumbens*, *Echinochloa crus-galli*, *Elytrigia repens*, *Eragrostis minor*, *Festuca altissima*, *Festuca pratensis*, *Humulus lupulus*, *Chenopodium album*, *Chenopodium hybridum*, *Lolium perenne*, *Panicum miliaceum*, *Phleum pratense*, *Phragmites australis*, *Plantago media*, *Plantago lanceolata*, *Plantago major*, *Poa nemoralis*, *Rumex crispus*, *Rumex obtusifolius*, *Sambucus nigra*, *Setaria viridis*, *Tilia cordata,* and *Urtica dioica*. The annual species are typical of ruderal sites and occur namely on the actively used landfill parts. Production of allergenic pollen in them depends on disturbances (waste deliveries) in their near surroundings. Some of individuals of these species may be damaged or destroyed by human activities on the landfill. In such places, their controlled regulation does not occur and if the plants are not killed by the process of landfilling, they can continue growing and producing considerable amounts of allergenic pollen.

**Figure 5:**
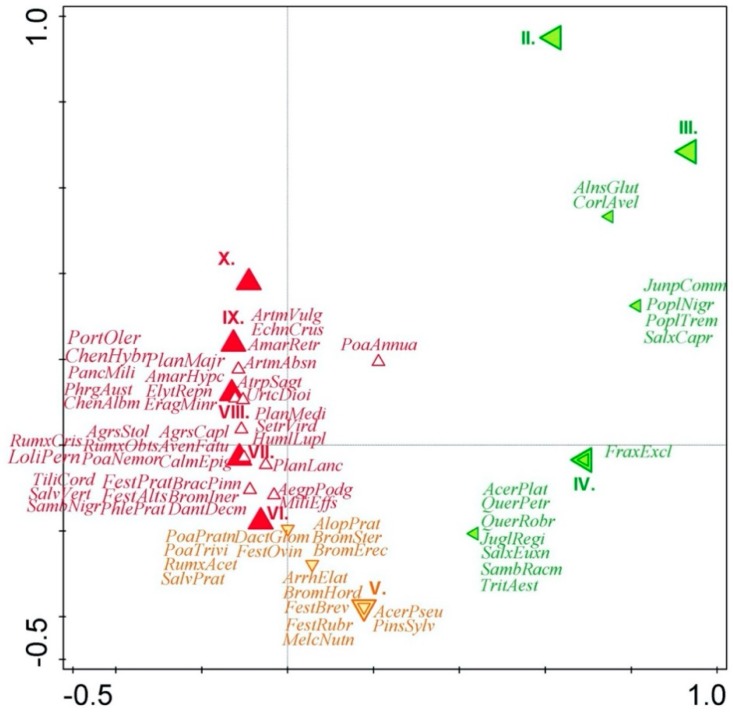
The relation between some plant species producing allergenic pollen and months of blooming.

**Figure 6:**
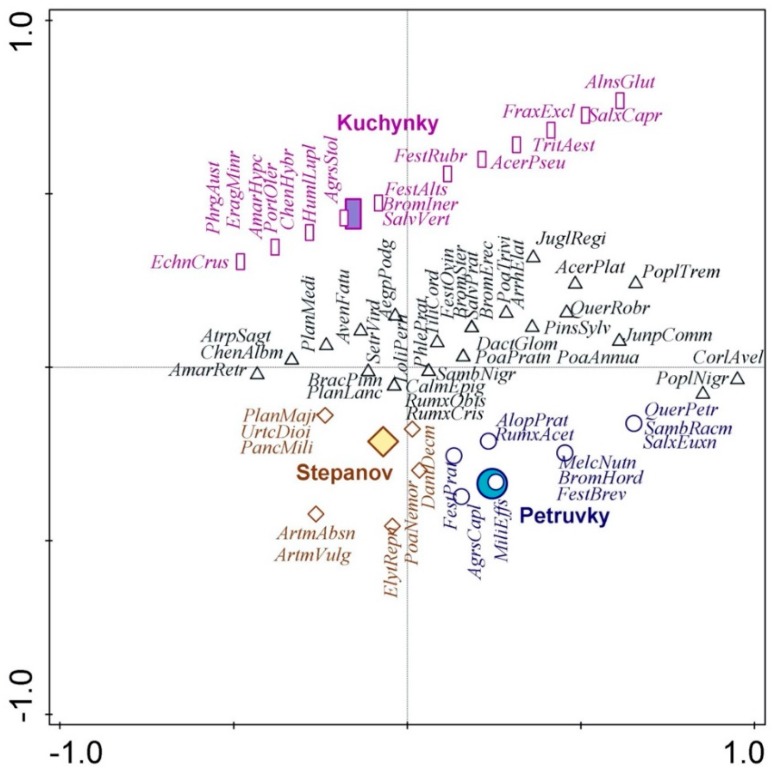
The relation between some plant species producing allergenic pollen and landfill locality.

Based on the CCA and Monte Carlo test, we created ordination diagrams assessing separately the relationships of detected species and their blooming times (Figure 5) and the landfill locality (Figure 6). Table 3 presents statistical significance and explained variations according to the evaluated factors.

Based on the statistical evaluation, we can state that the studied months of blooming exhibit a highly conclusive relationship with the species composition of studied vegetation. Figure 5 shows that in the period from February to April, the allergenic pollen from the landfills will be produced by the first group of species (green), in May the allergenic pollen will be produced by the second group of species (orange), and the third group of species (red) will produce allergenic pollen in the period from June to October.

The statistical evaluation revealed that the effect of landfill locality is also highly conclusive. Plant species occurring in greater abundance on the Kuchyňky site are plotted in violet color while plant species abundant on the Petrůvky and Štěpánovice sites are blue and brown, respectively. Black color is used for the typical plant species common to all studied sites, which produce allergenic pollen.

We also evaluated the year of monitoring. In this parameter, the results were statistically inconclusive, and this is why they are not presented graphically. The inconclusive differences between the years point to steady-state species composition and thus we can state that landfills may be the long-term source of allergenic pollen.

Generally, some research on air pollution from MSW landfill sites exists [40,41,42,43,44,45]; However, research tracking the allergenic pollen on MSW landfill sites is lacking. Mapping of allergenic pollen is practiced worldwide. According to McInnes et al. [46], allergenic pollen is produced by a number of trees, grasses, and weeds. In their study, the authors point out that exposure to such pollen grains can result in exacerbation of pollen-related asthma and allergenic conditions such as allergic rhinitis [47]. The authors also emphasize that currently it is very important to map the vegetation of plants with allergenic pollen to help affected individuals in the self-management of their allergy or asthma. Skjøth et al. [48] note that in Europe, grass pollen is the most important pollen allergen due to its massive distribution. Results of our research indicate that the vegetation spectra on the monitored MSW landfills are similar and that the identified plant species represent a significant source of the production of allergens. Particularly, the species blooming in the period from summer to autumn are common for all studied localities.

The results also show that the active landfill body houses plant species producing allergenic pollen. These species are particularly from the family of *Amaranthaceae*. In order to reduce the production of allergenic pollen of these plant species, it is necessary to change the management of landfilling. Firstly, the occurrence and development of vegetation on active landfills have to be monitored in the period from June to September. If these species are detected, the sites of their occurrence should be reduced either by cutting or by mechanical liquidation of plant stands. Sofiev et al. [49] noted that dispersion of pollen grains once they are released from a plant is dependent on many factors, predominantly meteorological. Land management is also important. For example, hay cutting can decrease amounts of pollen.

It is desirable that the vegetation composition is controlled on reclaimed landfills, which may help reduce the production of allergenic pollen. Part of landfill reclamation is also ecosystem restoration leading to severe losses and fragmentation of ecological habitats [50]. Such a newly created ecosystem has very specific functions and limitations [32,33]. Plant species have to provide for the expeditious coverage of the reclaimed surface and prevent soil erosion [9,10]. Further, their roots must not grow into the landfill body [38] and they should not produce large amounts of pollen. Plant species suitable for the biological reclamation of landfills are namely grasses [50], in which the formation of fertile suckers is time-limited, i.e., grass species blooming only in the first cut. Later, they form usually only vegetative offshoots, which do not flower and hence do not produce pollen. It is recommended to use grass mixtures with a low number of species and with the similar blooming period. From this point of view, suitable are for example *Arrhenatherum elatius*, *Festulolium*, and the like. Another important measure consists in the adaptation of the management of maintenance of reclaimed areas, especially the date of the first cut of grasses. The date of cut should be planned for the period of the phenological phase of the “end of earing” in order to prevent pollen formation.

It was conclusively shown in research conducted by Maiti et al. [51,52] and Ng et al. [53] that grass-legume or grass mixtures have a significant effect on the protection of soil and nutrient cycling as well as on water infiltration. Therefore, they can be used in the restoration of landfill sites. Moreover, grasses are growing fast, provide biomass, and have the ability to survive on the waste material, and are tolerant to adverse pH, extremely low nutrient conditions, and toxic metals. Extensive root system of these species holds loose soil particles and prevents soil erosion while enhancing productivity to a sustainable level [51,52]. During their growth, grasses are nitrogen intensive. For vegetation to fulfill its function in the restored ecosystem, it is advised to add representatives from the family of *Fabaceae* into the sowing mixture. Plant species from the family of *Fabaceae* are capable to supply atmospheric nitrogen and thus boost the growth of grasses sown together with them. At the same time, they are *entomophilous*, their pollen gets into the air only at limited amounts and this is why they are not considered as important producers of allergenic pollen. Examples of such species can be *Medicago lupulina*, *Lotus corniculatus,* etc.

Ecosystems of reclaimed landfills have their specific features [32,33]. The composition of plant mixtures used for the restoration of ecosystems of reclaimed landfills as well as the proportional representation of individual species in the mixtures should be subject to a more detailed scientific research.

Results obtained confirmed the hypothesis about MSW landfills being the sources of allergenic pollen. The results can be used for the formulation of changes in the management of waste landfilling, reclamation of landfills, and ecosystem restoration, which can reduce the production of allergenic pollen. Landfill restoration with vegetation has several benefits, including the creation of habitats for local wildlife [53,54,55,56,57]. However, evaluating the landfill vegetation from the view of the potential production of allergenic pollen, we can see that especially the ruderal plant species blooming during summer and autumn play an important role. These cosmopolitan species are represented in diverse areas and flourish on frequently disturbed ruderal sites. Their regulation is very difficult as well as eventual reduction of their producing allergenic pollen. This was confirmed in the study carried out by Kruczek et al. [58].

Health and life quality depend primarily on the physical, social, and economic environment in which humans live [59]. The environment is increasingly endangered by human activities such as landfilling [4,60] and there are only a few areas in the world that are not affected by them. The awareness about environmental pollution is increasing on an international scale [61]. Scientific approach is needed to determine the nature and extent of actual risks.

Landfill gas, leachates, wind-blown litter, insects, and rodents can represent a risk to human health and environmental quality. The authors state that the species composition of landfill vegetation can have a significant impact on air quality, namely in terms of the occurrence of strong allergens.

## 4. Conclusions

Allergenic pollen that produce vegetation on waste-landfill sites was evaluated and high pollen emissions were distinctly observed during spring and summer. Conversely, the landfill reclaimed by afforestation produced low or negligible pollen emissions. Landfills of MSW give space to vegetation, in which species producing allergenic pollen have a considerable representation. Pollen production depends on the blooming term of the given plant species. The analysis of research results indicated that there are three such periods. Early spring shows the pollen production of woody plants and shrubs in particular, yet the species do not occur in abundance on the monitored landfill sites and this is why the pollen production is limited. The second period is late spring and early summer, which is typical for the production of pollen by grasses and occur particularly on the reclaimed landfill parts. Thanks to cutting, their blooming time and hence pollen production can be easily controlled. To minimize the occurrence of allergenic pollen, the authors recommend cutting of reclaimed landfills in May and implementing the measure in the normative and legislative environment. The conducted research also showed that reclamation of landfills by afforestation significantly contributes to enhance air quality and condition of the environment. The last period is late summer and autumn when ruderal vegetation is blooming. Regulation of ruderal species is very troublesome in the environment of landfills and is usually not done in practice. This group includes plant species producing allergenic pollen, which should be focused on in order to develop technical procedures and normative regulations leading to the reduction of their incidence. Plant species with strongly allergenic pollen have a considerable share in the landfill vegetation. Thus, landfills are potentially significant sources of various kinds of allergenic pollen. Since the vegetation of landfills will produce allergenic pollen for a long time in the future, the problem should be given proper attention by botanists, environmentalists and physicians. The results of our research represent a good foundation for new studies of the phenomenon of allergenic pollen from MSW landfills and can be used to formulate a directive (norm) to protect human health. Landfill reclamation strategy and landfill cover have been scarcely studied so far.

## Figures and Tables

**Table 1:** Characteristics of municipal solid waste (MSW) landfill sites.

	Monitored Sites		
	Kuchyňky (C)	Petrůvky (B)	Štěpánovice (A)
Altitude (m a.s.l.)	270–285	550–650	450
Basin of River	Morava	Morava	Vltava
Type of landscape in the landfill surroundings	Intensively used agricultural landscape	Extensively used agricultural landscape	Landscape dominated by forests
Potential natural vegetation	Floodplain forests	Herb-rich beech forests	Acidophilous beech-fir-birch and pine oak forests

**Table 2:** Numbers of plant species found in the monitored sites, blooming time, and pollen evaluation.

Blooming Time and Significance of Pollen as Allergen		Monitored Sites		
		Kuchyňky (C)	Petrůvky (B)	Štěpánovice (A)
**Total**		192	162	127
Number of plant species flowering in selected months	February (II)	3	2	2
	March (III)	13	13	9
	April (IV)	32	43	27
	May (V)	92	93	62
	June (VI)	136	112	91
	July (VII)	148	117	105
	August (VIII)	125	89	88
	September (IX)	94	60	66
	October (X)	41	26	33
Plant species	Plant species producing allergenic pollen	45	40	29
	Wind-fertilized plant species not belonging to strong allergens	7	9	5
	Entomophilous plant species whose pollen gets into the air only at limited extent	139	112	92
	Plant species with no pollen production (cryptogams)	1	1	1

**Table 3:** Statistical significance of the evaluated factors (results of Monte Carlo test, CCA).

Factors	Explained Variation (%)	Statistical Significance (*p*-Value)	Pseudo F
Month of blooming	22.6	0.001	2.0
Landfill locality	23.0	0.001	3.6
Year of evaluation	6.0	0.2	8.4

## Appendix A

Plant species: *AcerPlat—Acer platanoides, AcerPseu—Acer pseudoplatanus, AgrsCapl—Agrostis capillaris, AgrsStol—Agrostis stolonifera, AlnsGlut—Alnus glutinosa, AlopPratn—Alopecurus pratensis, AmaHypc—Amaranthus hypochondriacus, AmaRetr—Amaranthus retroflexus, ArrhElat—Arrhenatherum elatius, ArtmAbsn—Artemisia absinthium, ArtmVulg—Artemisia vulgaris, AtrpSagt—Atriplex sagittata, AvenFatu—Avena fatua, BracPinn—Brachypodium pinnatum, BromErec—Bromus erectus, BromHord—Bromus hordeaceus, BromIner—Bromus inermis, BromSter—Bromus sterilis, CalmEpig—Calamagrostis epigejos, CorlAvel—Corylus avellana, DactGlom—Dactylis glomerata, DantDecm—Danthonia decumbens, EchnCrus—Echinochloa crus-galli, ElytRep—Elytrigia repens, EragMino—Eragrostis minor, FestAlts—Festuca altissima, FestBrev—Festuca brevipila, FestOvin—Festuca ovina, FestPrat—Festuca pratensis, FestRubr—Festuca rubra, FraxExcl—Fraxinus excelsior, HumlLupl—Humulus lupulus, ChenAlbm—Chenopodium album, ChenHybr—Chenopodium hybridum, JuglRegi—Juglans regia, JunpComm—Juniperus communis, LoliPern—Lolium perenne, MelcNutn—Melica nutans, MiliEffs—Milium effusum, PancMili—Panicum miliaceum, PhlePrat—Phleum pratense, PhrgAust—Phragmites australis, PinsSylv—Pinus sylvestris, PlanMedi—Plantago media, PlanLanc—Plantago lanceolata, PlanMajr—Plantago major, PoaAnnua—Poa annua, PoaNemor—Poa nemoralis, PoaPratn—Poa pratensis, PoaTrivi—Poa trivialis, PoplNigr—Populus nigra, PoplTrem—Populus tremula, QuerPetr—Quercus petraea, QuerRobr—Quercus robur, RumxAcet—Rumex acetosella, RumxCris—Rumex crispus, RumxObts—Rumex obtusifolius, SalxCapr—Salix caprea, SalxEuxn—Salix euxina, SambNigr—Sambucus nigra, SambRacm—Sambucus racemosa, SetrVird—Setaria viridis, TiliCord—Tilia cordata, TritAest—Triticum aestivum, UrtcDioi—Urtica dioica*.
